# Organellar-genome analyses from the lycophyte genus *Isoetes* L. show one of the highest frequencies of RNA editing in land plants

**DOI:** 10.3389/fpls.2024.1298302

**Published:** 2024-03-12

**Authors:** Jovani Bernardino de Souza Pereira, Renato Renison Moreira Oliveira, Santelmo Vasconcelos, Mariana Costa Dias, Cecílio Frois Caldeira, Dietmar Quandt, Guilherme Oliveira, Jefferson Prado

**Affiliations:** ^1^ Instituto de Pesquisas Ambientais (IPA), São Paulo, Brazil; ^2^ Bonner Institut für Organismische Biologie (BIOB), Abt. Biodiversität der Pflanzen, Rheinische Friedrich-Wilhelms-Universität Bonn, Bonn, Germany; ^3^ Instituto Tecnológico Vale, Belém, Brazil; ^4^ Departamento de Ciências Fisiológicas Universidade Federal do Amazonas, Manaus, Brazil

**Keywords:** evolutionary adaptation, lycophytes, mitogenome, plastome, protein-coding regions, RNA modification, vascular seed-free plants

## Abstract

RNA editing is a post-transcriptional process that challenges the central dogma of molecular biology by modifying RNA sequences, introducing nucleotide changes at specific sites, and generating functional diversity beyond the genomic code, especially when it concerns organellar transcripts. In plants, this phenomenon is widespread, but its extent varies significantly among species and organellar genomes. Among land plants, the heterosporous lycophytes (i.e., *Isoetes* and *Selaginella*) stand out for their exceptionally high numbers of RNA-editing sites, despite their morphological stasis and ancient lineage. In this study, we explore the complete set of organellar protein-coding genes in the aquatic plant group *Isoetes*, providing a detailed analysis of RNA editing in both the mitochondrial and plastid genomes. Our findings reveal a remarkable abundance of RNA editing, particularly in the mitochondrial genome, with thousands of editing sites identified. Interestingly, the majority of these edits result in non-silent substitutions, suggesting a role in fine-tuning protein structure and function. Furthermore, we observe a consistent trend of increased hydrophobicity in membrane-bound proteins, supporting the notion that RNA editing may confer a selective advantage by preserving gene functionality in *Isoetes*. The conservation of highly edited RNA sequences over millions of years underscores the evolutionary significance of RNA editing. Additionally, the study sheds light on the dynamic nature of RNA editing, with shared editing sites reflecting common ancestry whereas exclusive edits matching more recent radiation events within the genus. This work advances our understanding of the intricate interplay between RNA editing, adaptation, and evolution in land plants and highlights the unique genomic features of *Isoetes*, providing a foundation for further investigations into the functional consequences of RNA editing in this enigmatic plant lineage.

## 1 Introduction

RNA editing is a post-transcriptional process that alters RNA sequences through specific enzymatic reactions, which insert, delete, or substitute nucleotides at specific sites of RNA transcripts (Takenaka et al., 2013). RNA editing has challenged the central dogma of molecular biology that emphasizes how genetic information passes faithfully from DNA to RNA to proteins by creating RNA products that differ from their DNA templates (Knoop, 2011). Predominantly occurring at the first or second positions of codons, RNA editing usually alters the codon given in the genome (e.g., Grewe et al., 2011; Villarreal et al., 2018; Fauskee et al., 2021). This alteration leads to differences between the mature RNA and the genomic DNA-encoded codons, impacting the amino acids specified. Consequently, to predict the final protein sequence from a gene necessitates consideration of the mature RNA sequence, as the genomic sequence alone is insufficient for this purpose (Takenaka et al., 2013). Therefore, characterizing the abundance of RNA editing sites among species can help to understand the potential level of genic expression variation that ultimately may contribute to species adaptability.

Although RNA editing is widespread in land plants and highly frequent in the organellar genomes, its frequency varies greatly among different groups and between the plastome and chondrome (herein named mitogenome). In angiosperms, the known number of RNA-editing sites in the plastome ranges from 23 sites in *Cucumis melo* L. (Guzowska-Nowowiejska et al., 2009) to 184 sites in *Amborella trichopoda* Baill (Ishibashi et al., 2019). In the mitogenome, the range extends from 313 editing sites in *Populus* L. (Edera et al., 2018) to a substantial number of 902 sites in *Pulsatilla patens* (L.) Mill (Szandar et al., 2022). Among gymnosperms, edits in the plastome can vary from a complete absence in *Welwitschia mirabilis* Hook. f. (Fan et al., 2019) to 255 editing sites in *Ginkgo biloba* L (He et al., 2016), while the mitogenome range from 99 in *W. mirabilis* (Fan et al., 2019) to an impressive number of 1,240 sites in *Dioon* Lindl (Wu and Chaw, 2022). Notably, seed-free plants exhibit an even wider range of variation in RNA-editing sites. In plastomes, edits are surprisingly absent in the complex thalloid liverworts (Marchantiidae) (Rudinger et al., 2008), but reach the extreme number of almost 3,500 sites in *Selaginella uncinata* (Desv. ex Poir.) Spring (Oldenkott et al., 2014). In mitochondria, the limited available data suggest that the total number of edits ranges from 885 sites in *Salvinia cucullata* Roxb. (Li et al., 2018) to 1,782 in *Isoetes engelmannii* (Grewe et al., 2011) and 2,139 sites in *S*. *moellendorffii* Hieron (Hecht et al., 2011).

To date, most studies have examined differences in the abundance of RNA editing among major lineages (i.e., above the family level), although the variation within congeneric species or across multiple genera within the same family has also been under investigation. Additionally, studies have revealed a high variation in the abundance of RNA-editing events in distinct groups of closely related species. For instance, in the plastome, Kawabe et al. (2019) identified a difference of eight editing sites among three related species (and one subspecies) in *Arabidopsis* Heynh., whereas Fauskee et al. (2021) recorded a variation from 350 to 509 editing sites across three species of *Adiantum* L. Exceptionally high differences were even found in *Selaginella* Willk., with edits in the plastomes ranging from 720 to 3,494 sites in three species (Smith, 2020). As for the mitogenome, it usually shows higher RNA editing frequency than the plastome (Takenaka et al., 2013). Nonetheless, for the mitogenome, considerably less information is available concerning the extent of variation in RNA editing abundance among closely related taxa. Limited knowledge of RNA editing abundance for only a few species hinders our understanding of its variation among plants, particularly in closely related species, and the factors influencing it.

Interestingly, the heterosporous lycophytes, *Isoetes* and *Selaginella*, form a clade that exhibits the highest numbers of RNA-editing events in the organellar genomes among land plants. Specifically *Isoetes* comprises a globally distributed aquatic plant group with species inhabiting a wide range of climatic zones. The genus represents the only extant members of *Isoetales*, with fossil records extend back 350 million years to the Devonian period (Pigg, 1992). Notably, *Isoetes* displays a remarkable degree of morphological and genetic stasis, with its modern forms highly resembling their ancient relatives from the Triassic and Late Jurassic (Pigg, 1992; Hoot and Taylor, 2001). However, this stasis surprisingly contrasts with the exceptionally high number of mitochondrial RNA-editing sites observed in the genus (Grewe et al., 2011). The changes in the genetic information encoded by RNA through editing raise intriguing questions about the contribution of these edits to increase genic expression diversity within the genus, warranting further investigation.

With the advent of next-generation sequencing (NGS), our ability to identify RNA editing has greatly increased via matching high-throughput DNA and RNA sequencing. By cross-checking genomic and transcriptomic data, we can identify and characterize RNA-editing events, shedding light on the landscape of the RNA editome in closely related plant species.

In this study, we assembled the complete set of organellar protein-coding genes from distinct phylogenetic and operational taxonomic units (OTU) in *Isoetes* and addressed the abundance of RNA editing in the organellar genomes comparing genomic and transcriptomic data. We aimed to assess the extent of variation in the abundance of RNA editing within the genus to contribute to our understanding of RNA-editing landscapes in the land plants. We revealed the dual role of RNA editing in conserving functional proteins and enhancing encoded RNA diversity in *Isoetes*, providing valuable insights into these potentially adaptive mechanisms of *Isoetes*.

## 2 Materials and methods

### 2.1 Sampling

We selected *Isoetes cangae* J.B.S. Pereira et al., *I. echinospora* Durieu, and *I. taiwanensis* De Vol to estimate the RNA editing frequency and patterns. These taxa were selected because they are diploids representing the distinct major clades within *Isoetes*, and at the same time span the ecological and time dimension as they occur in distinct geographically habitats with different climatic conditions. Raw Illumina cDNA reads were produced for *I. cangae* in this study (**Supplementary File 1**, **Table S1**). Whereas raw data of Illumina RNA-Seq data of *I. echinospora* and *I. taiwanensis* were obtained from Hetherington et al. (2020) and Wickell et al. (2021), respectively (**Supplementary File 1**, **Table S1**). In brief, plants of *I. echinospora* were collected in Loch Aisir and Loch Dubhaird Mor, North West Sutherland, Scotland (Hetherington et al., 2020). Spores of these plants were sterilized and germinated under axenic conditions to ensure RNA purity. Total RNA was then extracted from root, corm, and leaf tissues obtained from approximately 50 plants, with each tissue type processed with multiple replicates. RNA extraction was carried out using the RNeasy Plant Mini Kit (Qiagen, USA), incorporating on-column DNase I treatment and with selective elimination of tRNA and rRNA according to the manufacturer’s instructions (see Hetherington et al., 2020). As for *I. taiwanensis*, RNA-Seq of different replicates came from five individuals from its single known natural living population in Northern Taiwan (Wickell et al., 2021), with DNA-seq also originating from multiple individuals from the same population but different individuals from RNA ones (personal communication with Li-Yaung Kuo). Although potential polymorphisms may arise due to such pooled sampling, we can assume genetic homogeneity within this small population, sharing the same plastome and mitogenome haplotypes. RNA libraries for *I. taiwanensis* were prepared using ribo-free kits, preceded by DNAse treatment to minimize contamination (personal communication with Li-Yaung Kuo). Raw data reads were downloaded from the NCBI Sequence Read Archive of the BioProject PRJNA438492 for *I. echinospora* and PRJNA735564 for *I. taiwanensis*. Besides these species, *I. serracarajensis* J.B.S. Pereira et al., *I. eludens* J.P. Roux, *I. longissima* Bory & Dur., *I. taiwanensis*, and *I. malinverniana* Ces. & De Not. were included in our dataset to address the diversity of the protein-coding gene structure and content in the mitogenomes (**Supplementary File 1**, **Table S2**).

### 2.2 Organellar-genomes assembly and annotation

The NextSeq500 Illumina platform was used for whole genome sequencing. Briefly, paired‐end libraries (2x 150 bp) were constructed from ~50 ng of genomic DNA, extracted following the procedures described in Nunes et al. (2018). Samples were subjected to a step of enzymatic and random fragmentation in which the DNA was simultaneously fragmented and bound to adapters using the QXT SureSelect kit (Agilent Technologies, Santa Clara, USA) according to the manufacturer’s instructions. The fragmented DNA was purified and subjected to an amplification step using primers complementary to the adapters and bound indexes. After libraries were quantified using the Qubit^®^ 3.0 Fluorimeter (Life Technologies, USA) and fragments were checked for size in the 2100 Bioanalyzer using the High Sensitivity DNA kit (Agilent Technologies, Santa Clara, USA). Then, libraries were diluted to loading concentration, pooled, and denatured. The sequencing run was performed using the NextSeq 500 v2 kit high‐output (300 cycles). *Isoetes taiwanensis* was sequenced using Illumina NovaSeq 6000 in the genomic project of Wickell et al. (2021; GenBank accession SRR15005049).

For the long-read sequencing in a PacBio Sequel (Pacific Biosciences) platform, we prepared a SMRTbell library following the manufacturer’s protocol, and using total genomic DNA extracted from *I. cangae*, as mentioned above.

The *de novo* assembly of the mitogenomes and plastomes was performed using NOVOPlasty version 2.6.3 (Dierckxsens et al., 2017). Particularly, the plastome sequences of *I. cangae* and *I. echinospora* were obtained from our previous publications by Nunes et al. (2018) and Pereira et al. (2021a) (GenBank accession MG019394 and MK804474), respectively. Prior to assembly, we trimmed reads with base quality Phred < 20 and length < 100 bp, and filtered out the remaining reads with >20% low-quality bases (Phred < 20) using Fastx-Toolkit (http://hannonlab.cshl.edu/fastx_toolkit/). The NOVOPlasty config files were configured with the following parameters: insert size 300, read length 150, type chloro and mito (for the plastome and mitogenome, respectively), genome range 120–200 kbp, and k-mer 21–39.

For the mitogenome, we ran NOVOPlasty multiple times with distinct “seed input”, selecting among the exons, in an effort to retrieve the initially recorded 24 protein-coding genes (Grewe et al., 2009). To ensure that no protein-coding genes were accidentally missed in the assembly, we also took the precaution of mapping reads to each of the 42 genes that comprise the complete set of protein-coding genes in land plants (see Mower, 2020). The BBsplit tool from the BBMap package (Bushnell, 2014) was used for the read mapping of multiple gene references. If reads were retrieved for a specific gene, they were assembled in Geneious Prime 2021 (https://www.geneious.com), and the resulting sequence was used as input seed for the *de novo* assembly in NOVOPlasty that allowed to confirm its location in the mitogenome.

Additionally, we assessed the gene arrangements, orders, and content in the mitogenome of *Isoetes cangae* using PacBio long reads. For the assembly, we first selected the continuous long (CL) reads by mapping them to the mitochondrial genome of reference (*Isoetes engelmanii* A.Braun; Grewe et al., 2009) using BBduk with a *k-mer* of 31 (Bushnell, 2014). The selected CL reads were subsequently corrected using the correction module of Canu (Koren et al., 2017). After correction, reads were once again mapped to the reference mitogenome for accuracy checking using Minimap2 (Li, 2018). The mapped reads were then assembled with Canu, generating contigs that were used as references in the following iteration of mapping and assembly using the unmapped reads. When no read could be mapped, the iteration stopped and a final assembly was performed, generating the final contigs.

Reference annotation was carried out using Geneious Prime 2021 (https://www.geneious.com). For the annotation of the mitogenomes, we utilized the mitochondrial genome and transcriptome data published for *I. engelmannii* by Grewe et al. (2009), (2011) as the reference. As for the plastome, *I. cangae* was used as the reference in Geneious Prime 2021 (https://www.geneious.com). Additional manual corrections were made based on the transcriptome data, which revealed the correct positions of the start and stop codons.

### 2.3 RNA-Seq data acquisition

We obtained *Isoetes cangae* leaf samples from a prior experiment (Caldeira et al., unpublished). Briefly, sporelings of *I. cangae* were cultivated for 12 months at the Plant Growth Laboratory of Instituto Tecnológico Vale in Belém, Brazil. Leaf samples were collected, immediately flash-frozen in liquid nitrogen, and stored at -80 °C for subsequent RNA extraction. Total RNA extraction was performed using the RNeasy Mini Kit (QIAGEN, USA), following the manufacturer’s instructions. Selective exclusion of tRNA and rRNA was implemented, with DNase treatment incorporated to minimize DNA contamination. RNA quality and concentration were assessed using an Agilent 2100 Bioanalyzer (Agilent Technologies, Santa Clara, USA) and a Qubit^®^ RNA High Sensitivity Assay Kit (Thermo Fisher Scientific, USA). cDNA libraries were prepared using 100 ng of total RNA from each sample and the TruSeq Stranded mRNA library prep kit (Illumina, San Diego, CA, USA). Library quality was assessed with the Qubit^®^ DNA Broad-range Assay Kit (Thermo Fisher Scientific, USA) and the Agilent 2100 Bioanalyzer (Agilent Technologies, Santa Clara, USA). Paired-end sequencing was conducted on an Illumina NextSeq 500 (San Diego, CA, USA) using a High Output kit (300 cycles).

### 2.4 Analysis of RNA editing

We initially performed quality trimming of cDNA raw reads, following the same procedure used for DNA sequencing. This step involved removing the remaining Illumina adaptors and low-quality tails. To handle the large amount of data obtained from Illumina RNA-Seq, we extracted organellar RNA reads using the BBMap (Bushnell, 2014). The extraction process was carried out using plastomes and mitochondrial protein-coding genes as references. Subsequently, cDNA reads were mapped to the organellar protein-coding genes using the BBMap (Bushnell, 2014) with a normal sensibility option in Geneious. For calling the consensus sequence, we chose the recommended highest quality (60%) threshold that also takes into account the relative quality scores for each base for the calculation. The variant finder in Geneious Prime 2021 (https://www.geneious.com) was used to identify DNA : RNA mismatches in the data. We reported all DNA: RNA mismatches, including putative cytidines to uridines sites (C‐to‐U or canonical RNA editing), putative uridines to cytidines sites (U‐to‐C or so‐called reverse editing) as well as other types of mismatches attributable to errors rather than RNA editing (A:G and G:A). For each reference genome position, we calculated the read depth of the transcriptome mapping and the proportion of reads containing a mismatched nucleotide.

There is a trade-off between false positive and false negative DNA–RNA mismatches in estimates of the amount of RNA editing as highlighted by Guo et al. (2015). Normally, read trimming reduces the number of non-editing mismatches (false positives), though it does not entirely eliminate such sites. On the other hand, stringent read trimming increases the rate of false negatives. To address these issues and strike a balance, we retained mismatches using the relatively least stringent threshold of 4.6% variant frequency requiring a minimum of three reads supporting the identified mismatch to minimize false negatives in our approach. We also manually examined the retained mismatches and eliminated false positives based on defined criteria, such as DNA heteroplasmy and/or errors in the reference, transcriptome read errors, and mapping artifacts (see also Guo et al., 2015). To detect heteroplasmic regions and/or errors in the reference genomes, we identified ambiguous bases in the genome by mapping DNA reads from our plastid and mitochondrial genome sequencing projects onto the references using the mapping to reference and find variants with a minimum variant frequency of 0.25 options in Geneious Prime 2021 (https://www.geneious.com). DNA–RNA mismatches in genomic ambiguous sites were discarded. We also set a maximum variant (*p-value* 10–6) and a minimum strand-bias (*p-value* 10–5) in the variant found to reduce mismatches introduced by imperfect primer binding occurring within 6 bp of the ends of all mapped transcript reads. Mismatches near exon/intron junctions were examined to determine whether they resulted from the mismapping of spliced transcript reads onto the unspliced genomic sequence.

### 2.5 Nucleotide diversity and hydrophobicity changes

We calculated the nucleotide diversity of the encoded RNA and genomic DNA for the same set of species, which included *I. cangae*, *I. echinospora*, and *I. taiwanensis*. In particular, for the estimates of nucleotide diversity of the mitogenome, we also included *I. engelmannii*, which also presents cDNA (mature RNA) and DNA data available in the GenBank. Analyses of the nucleotide diversity were conducted separately for the DNA genomic coding regions and edited RNA sequences. Each region was individually aligned using MAFFT v7.450 (Katoh and Standley, 2013). The estimates of nucleotide diversity per site were carried out using DnaSP 6 (Rozas et al., 2017) and taking into consideration the Pi (π).

The hydrophobicity index was used following Monera et al. (1995) and Sereda et al. (1994) taking into consideration a pH = 7. In a protein, hydrophobic amino acids are likely to be located in the interior, whereas hydrophilic amino acids are likely to be in contact with the aqueous environment.

## 3 Results

### 3.1 Mitochondrial gene content and order

**Figure 1:**
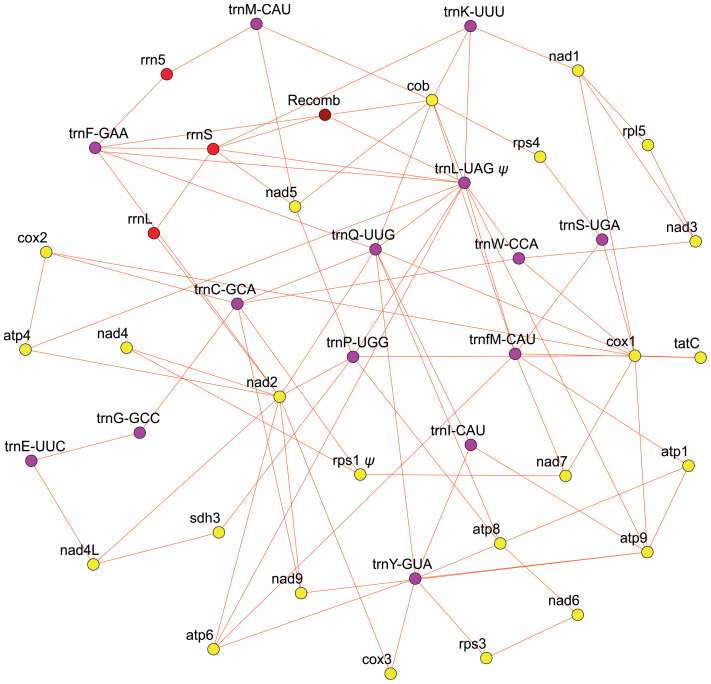
Diagram depicting the multiple arrangements of the mitochondrial genome of *Isoetes cangae*. Yellow, red, purple, and brown circles represent the protein-coding region, rRNA, tRNA, and recombination region, respectively.

Our effort to assess the mitogenome structure using PacBio long-read sequencing technology revealed a highly complex genomic structure in *Isoetes cangae* (**Figure 1**). Our PacBio assembly resulted in a total of 217 contigs with their sizes ranging from 993 to 88,323 base pairs. We recovered a total of 39 genes, with 23 protein-coding, 3 rRNA, and 13 tRNA in the mitogenome. Mitochondrial genes were found in different genomic environments, indicating a particularly high frequency of recombination events resulting in co-existing alternative gene arrangements and highly diverse gene orders in the genome. We also identified recombination breakpoints that make the physical existence of a potential master-circle encompassing the full mitochondrial genes highly unlikely.

**Figure 2:**
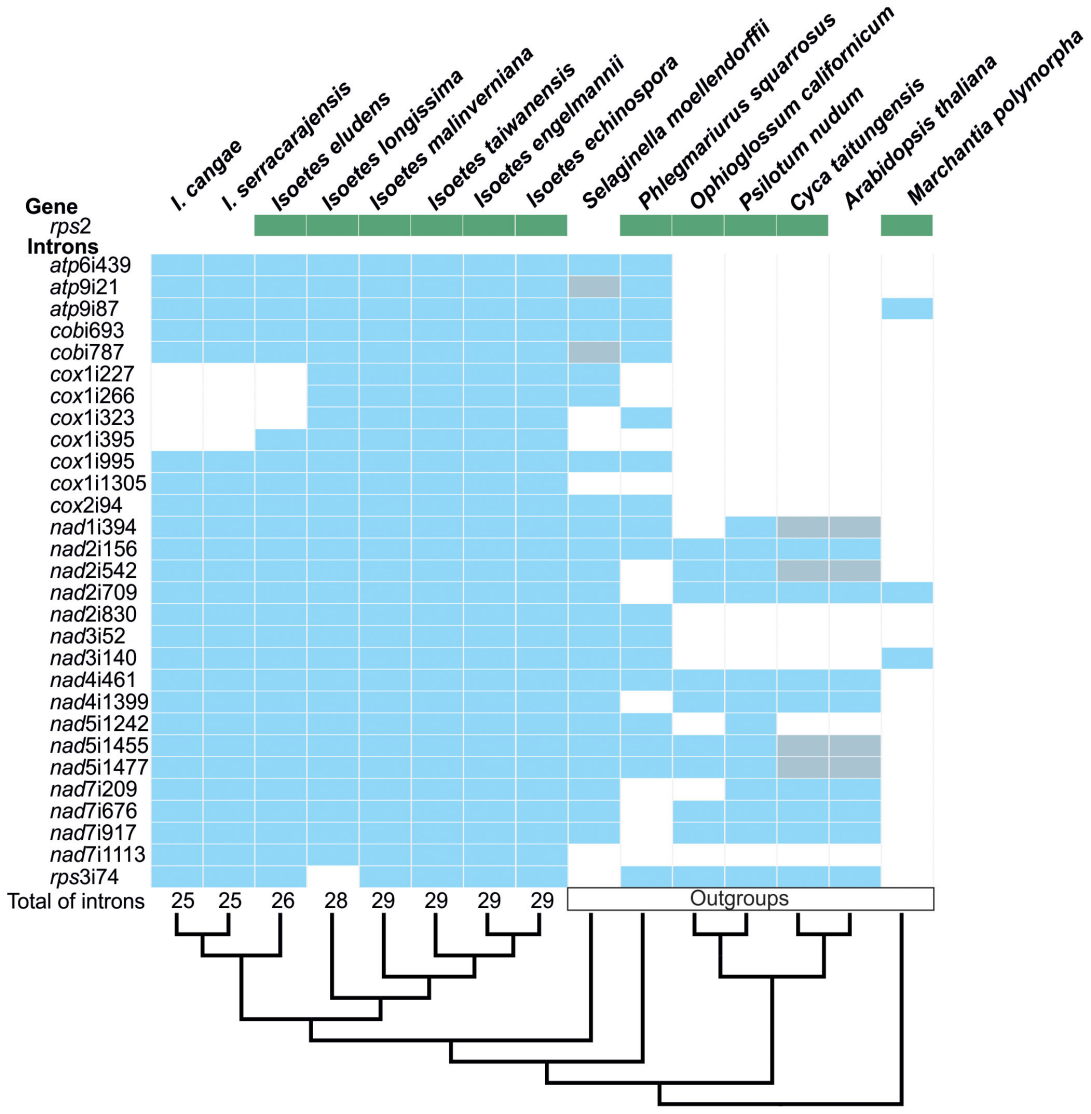
Mitochondrial intron content in protein-coding genes (blue) and *rps*2 gene distribution (green) in the genus *Isoetes* in comparison to its outgroups. Gray boxes represent pseudogenes corresponding to the introns. At the bottom, the cladogram shows the phylogenetic relationships among species, with the total number of introns indicated at the tips for *Isoetes*.

Using solely Illumina data, we were also able to retrieve a total of 23 mitochondrial protein-coding genes in *I. cangae* and *I. serracarajensis*. Conversely, *I. eludens*, *I. longissima*, *I. taiwanensis*, *I. malinverniana*, and *I. echinospora* exhibited a seemingly complete set of 24 genes, consistent with the findings reported in *I. engelmannii* by Grewe et al. (2009). Particularly, we noticed the absence of the *rps*2 gene in *I. cangae* and *I. serracarajensis* in comparison with the remaining species within the genus (**Figure 2**). RNA-Seq data also confirmed the absence of transcript for *rps*2 in *I. cangae*. Additionally, the full set of 29 introns documented for *I. engelmannii* (Grewe et al., 2009) was also observed in *I. taiwanensis*, *I. malinverniana*, and *I. echinospora* (**Figure 2**). However, we observed a reduction in the number of introns to 26 in *I. eludens* and 25 in *I. cangae*, and *I. serracarajensis*. This reduction is attributed to the substantial variation in the number of introns found in the *cox1* gene. Specifically, *I. eludens* possessed only three out of the six *cox1* introns, while *I. cangae* and *I. serracarajensis* were missing four out of the six identified *cox*1 introns. In the case of *I. longissima*, we observed the absence of the *rps*3i74 intron, although it was present in all other species within the genus (**Figure 2**). The discussion of plastome structure, gene content, and gene order was previously addressed by Pereira et al. (2021a).

### 3.2 The abundance of RNA editing in plastid and mitochondrial protein-coding genes

**Figure 3:**
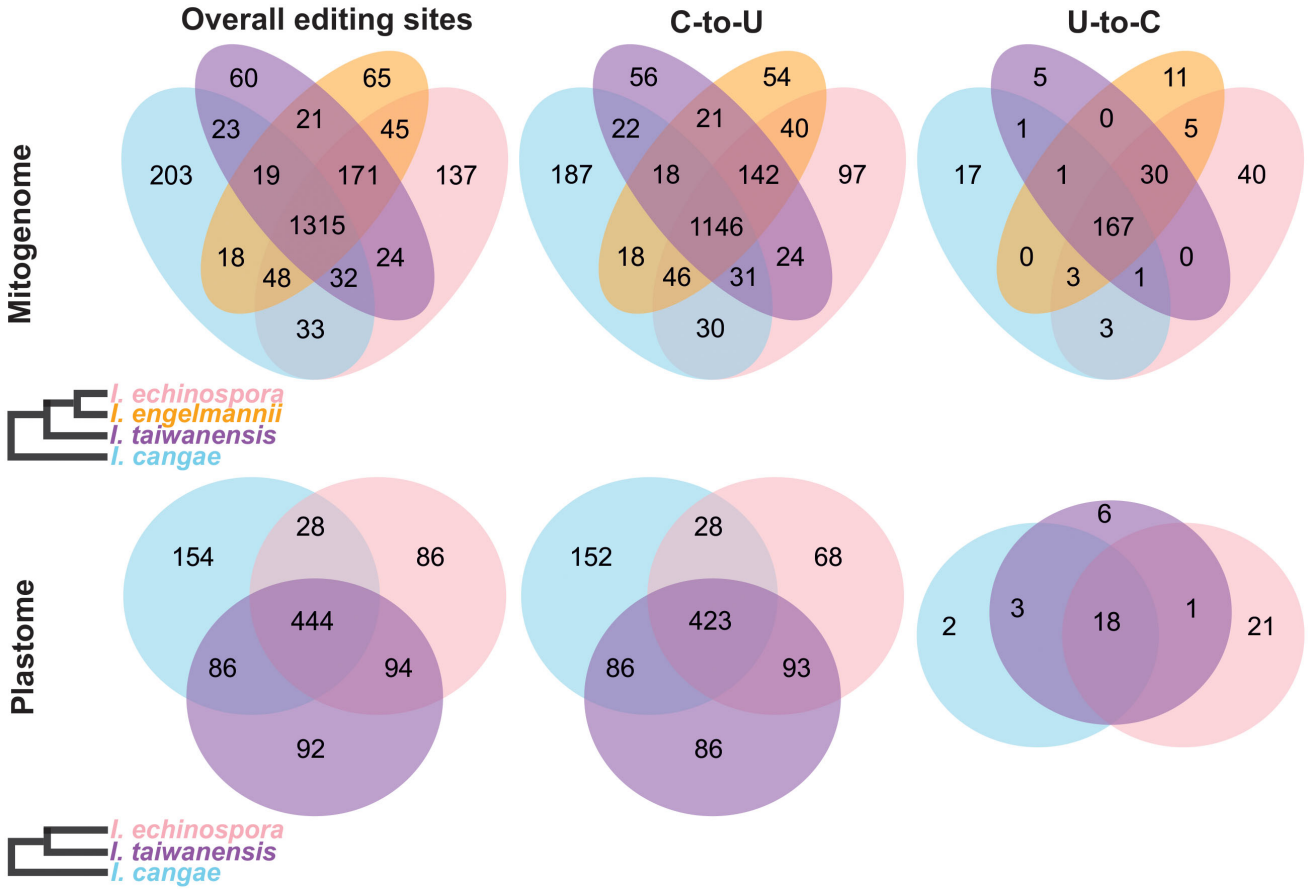
Venn diagram depicting shared and exclusive RNA-editing sites in the protein-coding regions of the mitogenome (top) and plastome (bottom) of *Isoetes*. On the left, cladograms show the phylogenetic relationships among the species. Mitochondrial data for *I. engelmanni* was obtained from NCBI, following the publication by Grewe et al. (2011).

Our RNA-editing analyses revealed a significant number of RNA editing both canonical (C-to-U) and reverse (U-to-C) in the mitogenome and plastome of *I. cangae*, *I. echinospora*, and *I. taiwanensis* (**Figure 3**). In the mitogenomes, a total of 1,691 RNA-editing sites were identified in *I. cangae*, 1,805 in *I. echinospora*, and 1,665 in *I. taiwanensis* (**Table 1**). These findings are consistent with the amount of 1,702 RNA-editing sites in the mitochondrial protein-coding regions of *I. engelmannii* (Grewe et al., 2011). Editing sites were recorded in all 24 mitochondrial protein coding-genes (**Supplementary File 2**). *nad*5 showed the highest number of edits, ranging from 149 in *I. taiwanensis* to 185 sites in *I. echinospora*. We also found that 1,315 (59.4%) mitochondrial edits were shared by all analyzed species. Additionally, we observed species-specific RNA-editing sites, with 33 unique sites in *I. cangae* and *I. echinospora*, 18 in *I. cangae* and *I. engelmannii*, 23 in *I. cangae* and *I. taiwanensis*, 45 in *I. echinospora* and *I. engelmannii*, 24 in *I. echinospora* and *I. taiwanensis*, and 21 in *I. engelmannii* and *I. taiwanensis* (**Figure 3**).

**Table 1:** Summary of mitochondrial and plastid RNA-editing abundances for three *Isoetes* species.

Changes	Mitogenome				Plastome		
	*I. cangae*	*I. echinospora*	*I. taiwanensis*	*I. engelmannii*	*I. cangae*	*I echinospora*	*I. taiwanensis*
Total of C -> U and U -> C	1,691	1,805	1,665	1,702	712	652	716
C -> U	1,498	1,556	1,460	1,485	689	612	688
U -> C	193	249	205	217	23	40	28
1st codon position	573	636	579	618	179	157	187
2nd codon position	804	867	814	851	400	391	414
3rd codon position	314	302	272	233	133	104	115
Start codon creation	12	12	12	12	22	23	25
Stop codon creation	9	9	9	9	6	6	6
Stop codon removal	78	85	83	85	16	15	19
Total of affected codon	1,453	1,564	1,448	1,479	652	610	670
Silent	220	211	187	154	99	79	95
Non-silet	1,233	1,353	1,261	1,325	553	531	575

In the plastome, we identified a total of 712 RNA-editing sites in *I. cangae*, 652 in *I. echinospora*, and 716 in *I. taiwanensis* (**Figure 3**; **Table 1**). RNA-editing sites were found in 73 out of 82 protein coding-genes (**Supplementary File 2**). *ndh*F revealed the highest number of edits in all species with their values number ranging from 62 in *I. echinospora* to 67 in *I. cangae*, whereas several genes showed only one editing site (**Supplementary File 2**). In *I. cangae*, for instance, *psb*D and *psb*N presented one editing site, however, RNA-editing events in these genes were absent in *I. echinospora* and *I. taiwanensis*. On the other hand, we found *psa*A with one edit in *I. echinospora* and *I. taiwanensis*, but no editing sites in *I. cangae*. Additionally, among the three species, 445 (45.2%) RNA-editing sites were shared by all three species (**Figure 3**). Furthermore, pair-wise comparisons on shared editing sites showed 28 unique edits in *I. cangae* and *I. echinospora*, 86 unique edits in *I. cangae* and *I. taiwanensis*, and 94 unique edits in *I. echinospora* and *I. taiwanensis* (**Figure 3**). The shared editing sites are consistent with the phylogenetic relationships among these species, with closely related species exhibiting the most shared sites.

The canonical C-to-U editing was much more frequent than the reverse U-to-C editing, both in the mitogenome and in the plastome (**Figure 3**; **Table 1**). However, in the mitogenome, the proportion of shared editing sites within *Isoetes* was fairly similar for both types of editing, with 59.4% (1146) for C-to-U and 58.9% (167) for U-to-C. In the plastome, 45.2% (423) of the C-to-U editing sites were shared among the species. This value was lower for the U-to-C sites with 35.3% (18). We also recorded several suspect events of RNA editing involving the conversion A-to-G and G-to-A (**Supplementary Files 3**, **4**) but they were not shared among the species and they are likely artifact errors of the sequencing.

**Figure 4:**
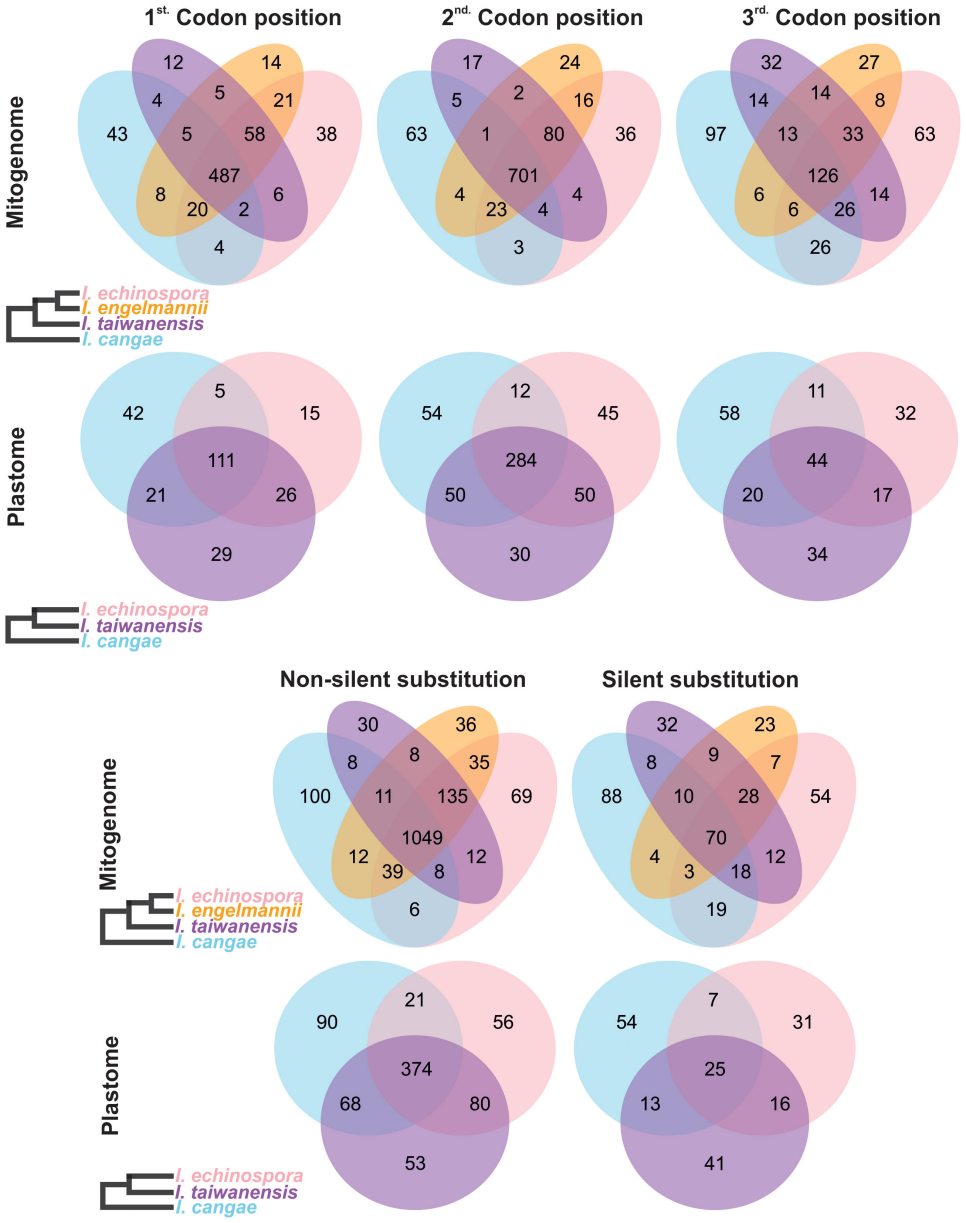
Venn diagram depicting specific types of shared and conserved RNA-editing sites in the protein-coding regions of the mitogenome and plastome of *Isoetes*. On the left, the cladograms show the phylogenetic relationships among the species. Mitochondrial data of *I. engelmannii* was obtained from NCBI following the publication by Grewe et al. (2011).

Regarding the codon position changes, the second position had the highest number of editing sites, followed by the first codon position, while the third position had the lowest number of editing sites in both organellar genomes (**Figure 4**; **Table 1**). When comparing the frequency of shared editing sites among the codon positions, changes in the second codon position were also more frequently shared among the species than in the first position. RNA-editing sites in the third codon position were not only less common but also less shared within *Isoetes*.

Most of the RNA-editing sites appear to have an effect on the protein, resulting in changes in the amino acid of the encoded protein (non-silent substitutions). The number of non-silent substitutions varied among the species, ranging from 1,233 in *I. cangae* to 1,353 in *I. echinospora* in the mitogenome, and from 531 in *I. echinospora* to 579 in *I. taiwanensis* in the plastome (**Figure 4**). In contrast, the number of silent substitutions was low, ranging from 154 in *I. engelmannii* to 220 in *I. cangae* in the mitogenome, and from 79 in *I. echinospora* to 99 in *I. cangae* in the plastome. Non-silent substitutions were also more conserved than silent ones, with a higher proportion of non-silent substitutions being shared among the three species (67.3% for the mitogenome and 50.4% for the plastome) compared to silent substitutions (18.2% for the mitogenome and 13.4% for the plastome) (**Figure 4**).

We also tested the hypothesis that the abundance of RNA editing can be predicted by the GC content of the plastid protein-coding regions (**Supplementary File 5**). As neither the GC content nor RNA-editing events in protein-coding regions showed a non-normal distribution in land plants (Shapiro-Wilk test, *p-value* < 0.05), we calculated Kendall’s tau correlation coefficient, which showed that GC% cannot reliably predict the abundance of RNA editing in plants (correlation coefficient = 0.26; *p-value* = 0.129). Nevertheless, within the genera, linear regression analysis showed a highly significative correlation between the GC% and the editing (R2 = 0.42; *p-value* = 0.003) (**Supplementary File 5**).

### 3.3 Hydrophobicity conversions and nucleotide diversity and

**Figure 5:**
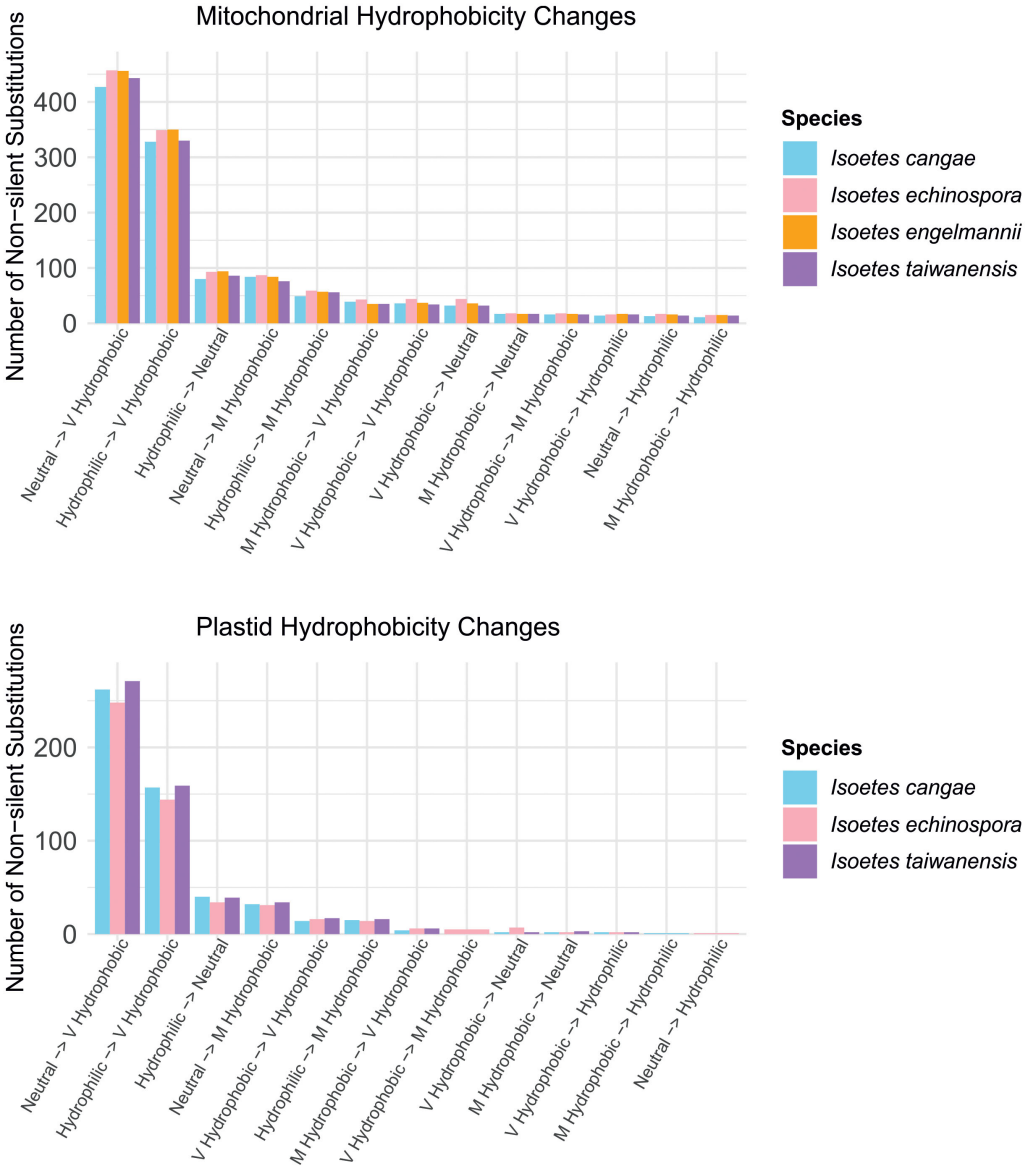
Comparisons of the number of non-silent substitutions that lead to changes in hydrophobicity of the amino acids via RNA editing in the mitochondrial (top) and plastid (bottom) protein-coding regions in *Isoetes*.

RNA editing leads to common patterns of amino acid hydrophobicity conversions in the encoded proteins in the mitogenome and plastome (**Figure 5**). The vast majority of non-silent RNA edits convert codons from neutral or hydrophilic amino acids to those that are highly hydrophobic. In contrast, a small proportion of edits involve conversions from very or moderately hydrophobic to neutral or hydrophilic amino acids, and even fewer changes conserve the hydrophobicity level of the amino acids.

**Figure 6:**
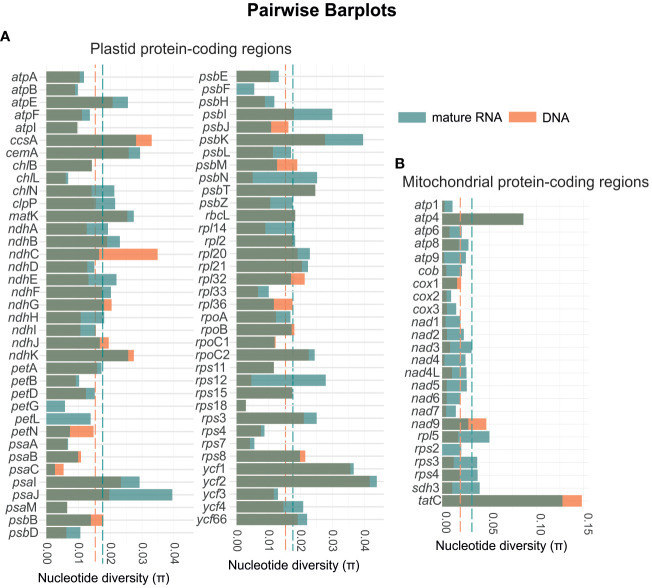
Diagram showing the variation in nucleotide diversity for the mature RNA (green bars) and genomic DNA (orange bars) for both the plastid **(A)** and mitochondrial genes **(B)**. The green and orange dashed lines represent the overall means of nucleotide diversity for the mature RNA and DNA, respectively. In the plastome, the mean nucleotide diversity increases from 0.015 in DNA to 0.018 in mature RNA. In the mitogenome, the mean nucleotide diversity rises from 0.019 in DNA to 0.032 in mature RNA.

In general, we also observed an increase in the nucleotide diversity from genomic DNA to mature RNA (**Figure 6**). The RNA of the mitochondria showed an average nucleotide diversity of 0.0316 which is higher than the average diversity of the genomic DNA which was 0.0187 (**Supplementary File 2**). Among the 24 coding-protein genes, *tat*C showed the highest nucleotide diversity with π = 0.1277 and 0.1484 for the RNA and genomic DNA, respectively. The gene *cox*2 showed the lowest nucleotide diversity (π = 0.0095) for the RNA and *rps*2 was the single region not to show diversity for the genomic DNA. Similarly, for the chloroplast, we found the highest average nucleotide diversity of 0.0177 for the RNA, while the average diversity of the genomic DNA was 0.0151. The gene *ycf*2 showed the highest nucleotide diversity of π = 0.0440 and 0.0418 for the RNA and genomic DNA, respectively. Whereas *psa*C revealed the lowest diversity of π = 0.0027 for the RNA, and *pet*G, *pet*L, and *psb*F present no nucleotide diversity in the analyzed species.

## 4 Discussion

*Isoetes* is an appealing focal group for studies of RNA editing because of its long timescale diverged from its sister group (*Selaginella*), high frequency of canonical (C-U) and reverse (U-C) RNA editing, and morphological and genetic conservation. This study marks the first infrageneric comparison of the mitochondrial editome in vascular seedless plants and stands out as one of the rare investigations conducted on the plastid editome.

*Isoetes* exhibits one of the highest frequencies of RNA-editing events in organellar-genomes among land plants with 1,665–1,805 edits in the mitogenomes. In fact, the number of edits in *Isoetes* surpasses the frequency of RNA editing found in other plant groups, second only to *Selaginella* (Hecht et al., 2011; Oldenkott et al., 2014; Smith, 2020). Organelle RNA editing in land plants is believed to be of monophyletic origin (Tillich et al., 2006), though it is unclear the extent of RNA-editing abundance of the ancestor. However, the high abundance of RNA editing reported in *Isoetes* and *Selaginella* is consistent with the presence of a common ancestor that already possessed abundant RNA-editing sites in the Devonian period when the splitting between these two groups likely took place (Kenrick and Crane, 1997; Klaus et al., 2017). Thus, the high frequency of RNA editing in *Isoetes* and *Selaginella* indicates that RNA editing is a time-long-preserved evolutionary mechanism within heterosporous lycophytes.

Our findings support the possibility that RNA editing could confer adaptive ecological advantages. Not only do RNA-editing events predominantly occur at the second and first codon positions, but edits at these positions are also more widely conserved within *Isoetes*. Additionally, we observed that RNA-editing events resulting in non-silent substitutions are more frequent and conserved than those leading to silent changes. These findings strongly suggest that RNA editing plays a crucial role in fine-tuning protein structure and function (see further), ultimately influencing the adaptation and evolution of *Isoetes*. Furthermore, the conservation of highly edited RNA sequences in the genomes of heterosporous lycophytes over an extended period underscores the role of evolutionary processes in preserving this post-transcriptional mechanism likely as an adaptive factor for heterosporous lycophyte plants. Furthermore, the prevalence of shared editing sites in both organellar-genomes suggests a common evolutionary origin and subsequent diversification of the edits, highlighting the dynamic nature of RNA editing in *Isoetes*.

The extent of variations in the abundance of RNA editing within *Isoetes* is relatively less pronounced than in ferns. In *Isoetes*, the differences in edits between *I. cangae* and the closely related species group, *I. taiwanensis* and *I. echinospora*, are two and 62, respectively. On the other hand, in *Adiantum*, the differences in RNA edits between *A. capillus-veneris* L. and the closely related species, *A. shastense* Huiet & A.R.Sm. and *A. aleuticum* (Rupr.) C.A.Paris, are 159 and 155, respectively (Fauskee et al., 2021). In addition, *Isoetes* species exhibit a higher proportion of shared editing sites than *Adiantum*. While it remains uncertain whether the extent of RNA-editing variation can be correlated with divergence ages within these genera, the most conserved RNA-editing landscapes in *Isoetes* align with the young age of the earliest divergence event among the analyzed species, estimated at approximately 20 million years ago based on plastome dating analyses (Pereira et al., 2021b). In contrast, the earliest age of divergence among *Adiantum* species was approximately 60 million years ago (Regalado et al., 2018; Fauskee et al., 2021).

Although the number of edits in the mitogenome surpasses 2.5-fold the frequency of RNA editing in the plastome, we found similar patterns in the editomes between them. The majority of edits are concentrated at the second codon position, followed by the first codon positions, C-to-U changes are more common than U-to-C changes, and non-silent substitutions are more commonly shared among the species. These patterns are commonly observed in other groups such as in the ferns (Guo et al., 2015, 2017; Fauskee et al., 2021), hornworts (Villarreal et al., 2018), gymnosperms (Chen et al., 2011; He et al., 2016) and angiosperms (Giegé and Brennicke, 1999; Edera et al., 2018). However, intriguingly, we identified distinct proportions of conservation for C-to-U and U-to-C edits between the mitogenome and plastome. While there was a slight difference in the proportion of shared edits between C-to-U and U-to-C in the mitogenome (59.3% and 58.8%, respectively), this difference is more pronounced in the plastome, with the respective shared edited sites being 45.5% for C-to-U and 35.3% for U-to-C.

One interesting aspect of our study is the number of 1,692–1,808 edits found using Illumina RNA-Seq is consistent with a previous study that reported 1,704 RNA-editing sites in the mitochondrial genes of *I. engelmannii* using Sanger sequencing (Grewe et al., 2011). As the different sequencing strategies reveal almost identical results, differences in the observed RNA-editing frequency are therefore independent of the sequencing methods used. However, there is a notable discrepancy in the number of RNA-editing events reported for the plastome of *I. taiwanensis* between the present study and the findings by Wickell et al. (2021) using the same dataset. We respectfully believe that this difference can be attributed to the stringent threshold of 10% utilized in the analysis by Wickell et al. (2021), which may have resulted in an increased number of false negatives, particularly at the second codon position where an anomalous occurrence of the lowest number of edits was observed. Our own experiment using a stringent threshold of 10% (instead the 4.6% as consider here) increased substantially the number of false negatives in *I. echinospora*, as evidenced by the retention of several internal stop codons in sites that clearly undergo RNA editing.

One critical aspect requiring attention in our analysis is the incorporation of multiple individuals in RNA sequencing datasets especially in *I. taiwanensis* (Wickell et al., 2021), potentially introducing the risk of false positive sites. This point demands careful consideration as it underscores the inherent difficulty in distinguishing genuine RNA-editing events from sequencing artifacts, particularly within datasets derived from multiple individuals. Furthermore, it is important to note that disparities in library preparation methods could further contribute to discrepancies in the observed RNA-editing frequencies across studies.

Regarding the impact of RNA editing on the encoded protein, our results suggest a consistent trend towards increased hydrophobicity in the encoded proteins after RNA editing, both in the mitogenome and plastome (**Figure 5**). This trend was also observed in other plant groups (as reviewed by Jobson and Qiu, 2008), such as angiosperms (Giegé and Brennicke, 1999; Ishibashi et al., 2019), the hornwort genus *Anthoceros* (Kugita et al., 2003), and in the ferns genus *Adiantum* (Fauskee et al., 2021). Organellar genomes are known to contain a relatively large proportion of genes encoding membrane-bound proteins that are rich in hydrophobic amino acids (Covello and Gray, 1993; Gray et al., 1999). The increased hydrophobicity of mitochondrial and plastid-encoded membrane-bound proteins in *Isoetes* reflect the preservation of hydrophobic residues, supporting the idea that RNA editing plays a role in conserving gene functionality in mitogenomes and plastomes, likely conferring a selective advantage.

It is worth noting that despite the lack of morphological variation within *Isoetes*, the genus remarkably presents adaptability to diverse habitats in a wide range of climatic zones on the planet. For instance, *I. cangae* occurs in tropical areas (Pereira et al., 2016), whereas *I. echinospora* is found in temperate to arctic regions. The presence of species-specific RNA-editing events likely affects the physicochemical properties of the proteins encoded by these sequences and the increase in nucleotide diversity, and thus, RNA editing might be involved in species-specific adaptations and functional diversification, enabling *Isoetes* to thrive in distinct aquatic habitats across the planet.

Surprisingly, the frequency of RNA editing in the plastid protein-coding regions within *Isoetes* is higher than the 581 reported edits in *Selaginella lepidophylla* (Hook & Grev.) Spring (Smith, 2020). Smith (2009) suggested a positive correlation between the GC content of organellar DNA of land plants and the abundance of organellar RNA editing. However, the usual GC content observed for *Isoetes* (37.6-38.1%; Nunes et al., 2018; Pereira et al., 2021a) compared to the highest GC content of 51.9% in *S. lepidophylla* (Smith, 2020), clearly does not match with the higher number of editing sites in *Isoetes*, in this case. Additionally, although we observed a correlation between GC% and the abundance of RNA editing within genera, it is important to note that this correlation is not a general assumption, as GC% cannot be used to predict the abundance of RNA editing across major plant groups (**Supplementary File 5**, **Figure S2**).

When it comes to the mitogenome, the comparative evaluation of the extent of variation of RNA-editing events is limited partially due to the scarcity of studies that demonstrate the complete set of mitochondrial protein-coding genes. This difficulty arises from the challenges associated with annotating mitochondrial genes, especially considering lineages extremely rich in RNA editing (Mower, 2020), and by rampant recombination (Gualberto and Newton, 2017).

We observed that PacBio long-read assemblies of the mitogenome of *I. cangae* provided not only evidence of the absence of master-circle encompassing the 39 identified genes, but also showed an extreme complex mitochondrial genomic structure with multiple gene arrangements. Unlike their metazoan and fungal counterparts, the mitochondrial master-circle is uncommonly found among plants mostly due to the presence of dispersed repeats that contribute to extensive homologous recombination (Stern and Palmer, 1984; Alverson et al., 2010; Gualberto and Newton, 2017).

*Isoetes* exhibits a reduction in the number of mitochondrial genes compared to other land plants (Mower, 2020), with a variation of 23-24 protein-coding genes. Little variation in the number of mitochondrial genes is also observed in *Selaginella*, with protein-coding genes varying from 17 in *S. nipponica* Franch. & Sav. (Kang et al., 2020) to 18 in *S. moellendorfii* (Hecht et al., 2011). Our PacBio assemblies not only contributed to the understanding about the diversity of gene and intron contents and genic arrangements in *Isoetes*, but also provided the basis to address the amount of RNA editing in the present study.

In conclusion, this study presents compelling evidence for the dual role of RNA editing in *Isoetes*. On one hand, it serves as an ancient evolutionary mechanism that has persisted over deep time, while on the other, it can acts as a modulator of species-specific adaptations. RNA editing plays a central role in shaping genetic expression and functional properties. Thus, we hypothesize that it contributes significantly to the diversity, evolution, and adaptability of *Isoetes* in various environmental conditions.

## Data availability statement

The datasets presented in this study can be found in online repositories. The names of the repository/repositories and accession number(s) can be found in the article/**Supplementary Material**.

## Author contributions

JBSP: Conceptualization, Data curation, Formal analysis, Investigation, Methodology, Validation, Writing – original draft, Writing – review & editing. RRMO: Formal analysis, Investigation, Methodology, Writing – review & editing. SV: Methodology, Writing – review & editing. MCD: Methodology, Writing – review & editing. CFC: Methodology, Writing – review & editing. DQ: Funding acquisition, Resources, Writing – review & editing. GO: Funding acquisition, Resources, Writing – review & editing. JP: Funding acquisition, Resources, Supervision, Writing – review & editing.

## Supplementary material

Supplementary File 1 SRA accessions of raw reads and GenBank accessions.

Supplementary File 2 Number of edits and nucleotide diversity per gene.

Supplementary File 3 Number of RNA-editing sites in the mitogenome of Isoetes.

Supplementary File 4 Number of RNA-editing sites in the plastome of Isoetes.

Supplementary File 5 Statistic tests on the correlation between CG content and number of edits in the plastome.
