# Supplementary material for: Organellar-genome analyses from the lycophyte genus Isoetes L. show one of the highest frequencies of RNA editing in land plants
Source: Front Plant Sci. 2024 Mar 12;15:1298302. doi: 10.3389/fpls.2024.1298302 (PMC10977995; doi:10.3389/fpls.2024.1298302)
Supplement: Supplementary File 5 — Statistic tests on the correlation between CG content and number of edits in the plastome. [file DataSheet_5.docx]

**SUPPLEMENTARY FILE 5.** Statistic tests on the correlation between CG content and number of edits in the plastome.

**Supplementary File 5, Table 1.** RNA-editing and GC content in protein-coding regions (CDS) of the plastome

| Species | GC content within CDS | Genbank accession | RNA-editing within CDS | Reference |
| --- | --- | --- | --- | --- |
| Adiantum aleuticum | 0.447 | MH173079 | 480 | Fauskee et al 2021 |
| Adiantum capillus-veneris | 0.416 | NC_004766 | 332 | Wolf et al 2004 |
| Adiantum shastense | 0.440 | MG432483 | 481 | Fauskee et al 2021 |
| Amborella trichopoda | 0.391 | NC_005086 | 165 | Ishibashi et al 2019 |
| Anthoceros formosae | 0.345 | AB086179 | 942 | Kugita et al 2003 |
| Cycas taitungensis | 0.393 | NC_009618 | 81 | Chen et al 2011 |
| Ginkgo biloba | 0.390 | AB684440 | 255 | He et al 2016 |
| Isoetes cangae | 0.379 | MG019394 | 712 | This study |
| Isoetes echinospora | 0.380 | MK804474 | 652 | This study |
| Isoetes taiwanensis | 0.381 | SRX11317221 | 716 | This study |
| Leiosporoceros dussii | 0.333 | MH577299 | 102 | Villareal et al 2018 |
| Ophioglossum californicum | 0.415 | KC117178 | 231 | Guo et al 2015 |
| Oryza sativa | 0.394 | NC_031333 | 33 | Ishibashi et al 2019 |
| Psilotum nudum | 0.360 | KC117179 | 24 | Guo et al 2015 |
| Selaginella kraussiana | 0.515 | MH549643 | 1,104 | Smith 2020 |
| Selaginella lepidophylla | 0.502 | NC_040927 | 581 | Smith 2020 |
| Selaginella uncinata | 0.543 | AB197035 | 3,415 | Oldenkott et al 2014 |
| Vitis vinifera | 0.379 | NC_007957 | 95 | Ishibashi et al 2019 |


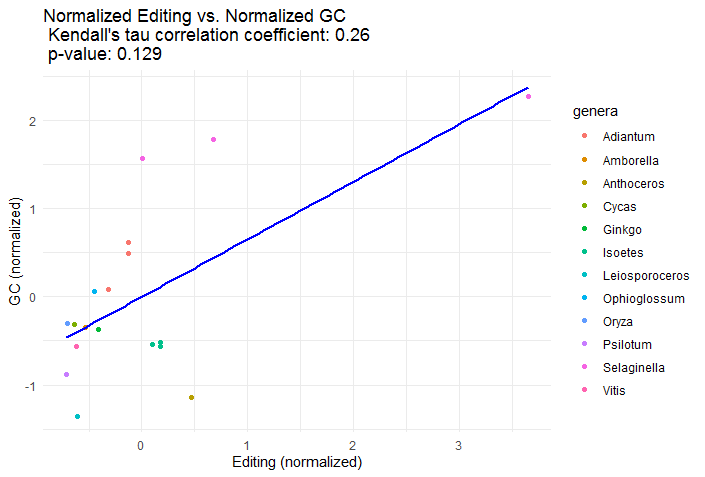


**Supplementary File 5, Figure 1.** Kendall's tau correlation coefficient between the GC% content and abundance of RNA editing in plants (correlation coefficient = 0.26; *p-value* = 0.129).


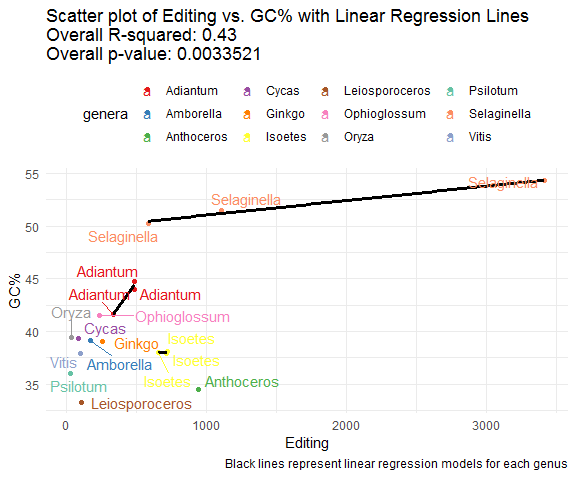


**Supplementary File 4, Figure 2.** Linear regression analysis showed a significative correlation between the GC% and the editing within genera in land plants (R2 = 0.42; *p-value* = 0.003).
